# Tobacco, cannabis, alcohol, and polysubstance use disparities among sexual identity groups of US young adult women and men

**DOI:** 10.1016/j.abrep.2024.100571

**Published:** 2024-11-23

**Authors:** Erin A. Vogel, Katelyn F. Romm, Carla J. Berg

**Affiliations:** a TSET Health Promotion Research Center, Stephenson Cancer Center, Univeristy of Oklahoma Health Sciences Center, Oklahoma City, OK, USA; b Department of Pediatrics, College of Medicine, University of Oklahoma Health Sciences Center, Oklahoma City, OK, USA; c Department of Prevention and Community Health, Milken Institute School of Public Health, George Washington University, Washington, DC, USA; d George Washington Cancer Center, George Washington University, Washington, DC, USA

**Keywords:** Alcohol, Cannabis, Polysubstance, Sexual minority, Tobacco, Young adult

## Abstract

**Background:**

Sexual minority young adults (SMYAs) display higher rates of polysubstance use (i.e., current use of multiple substances) than their heterosexual peers, but limited research has explored differences by gender and specific sexual identity.

**Methods:**

Latent class analyses (LCAs) examined past-month use of combustible tobacco (i.e., cigarettes, cigars, hookah), e-cigarettes, cannabis, and alcohol among 2,343 YAs (*M*_age_ = 24.69, SD = 4.70; 57.4 % women, 42.6 % men; 18.0 % bisexual, 9.4 % gay or lesbian; 36.2% racial/ethnic minority) residing in 6 US metropolitan areas. Multinomial logistic regressions examined associations among sexual identity (bisexual, gay/lesbian, heterosexual) and polysubstance use classes among women (*n* = 1,345) and men (*n* = 998), separately.

**Results:**

LCA yielded a 5-class solution: primarily-alcohol use (29.5%), polysubstance use (i.e., use of all 4 substances, 24.3%), non-use (18.1%), cannabis and alcohol co-use (16.3%), and e-cigarette, cannabis, and alcohol co-use (11.8%). Bisexual and lesbian (vs. heterosexual) women displayed lower odds of primarily-alcohol use, whereas bisexual (vs. heterosexual) women displayed higher odds of polysubstance use, as well as e-cigarette, cannabis, and alcohol co-use. Bisexual (vs. heterosexual) men displayed lower odds of primarily-alcohol use, whereas gay (vs. heterosexual) men displayed lower odds of polysubstance use, as well as e-cigarette, cannabis, and alcohol co-use.

**Conclusions:**

Bisexual women were at greatest risk for polysubstance use of combustible tobacco, e-cigarettes, cannabis, and alcohol, whereas bisexual and gay men were at lower risk than heterosexual men for polysubstance use. Results underscore the need for tailored interventions and campaigns to consider patterns of co-occurring tobacco, cannabis, and alcohol use, particularly for bisexual women.

## Highlights

• Young adults used tobacco, cannabis, and alcohol in several discernible patterns.
• Bisexual women were at risk for tobacco, cannabis, and alcohol polysubstance use.
• Bisexual and gay men were at lower risk for polysubstance use.
• Substance use interventions for bisexual women should consider co-use patterns.

## 1 Introduction

Young adulthood is a developmental period for forming behavioral patterns with long-term health implications (Harris, 2010), such as initiating or escalating substance use (Richmond-Rakerd et al., 2017), and coincides with increased independence and social pressures that may be conducive to substance use (Stewart et al., 2023). Young adults (YAs) aged 18–25 in the United States (US) have notably high prevalence of past-month (current) use of alcohol (50.1 %), nicotine or tobacco (24.7 %), and cannabis (24.1 %) (Substance Abuse and Mental Health Services Administration, 2023), conferring risks such as reduced engagement in school or work (Amato et al., 2021, Duckworth et al., 2023) and the development of substance use disorders (Substance Abuse and Mental Health Services Administration, 2023). Compared to single-substance use, polysubstance use of tobacco, cannabis, and alcohol in young adulthood may have additive risks, such as more alcohol-related and cannabis-related consequences (e.g., blackouts, risky sexual activity, verbal altercations, financial difficulties, legal problems) (Florimbio et al., 2023, Hayaki et al., 2016) and lower odds of tobacco cessation (Vogel, Rubinstein, Prochaska, & Ramo, 2018). Polysubstance use of tobacco, cannabis, and alcohol may elevate young adults’ risk for substance-related problems.

Young adulthood may be a particularly vulnerable time for sexual minority (SM) individuals, who may be developing, defining, and disclosing their sexual identity (Kaestle, 2019). Minority Stress Theory posits that SM individuals face unique minority stressors (e.g., discrimination, internalized stigma) related to their sexual identity, leading to greater mental health problems and associated use of substances to cope (Hatzenbuehler, 2009, Meyer, 2003). SMYAs indeed display disproportionately higher rates of tobacco, cannabis, and alcohol use (The Trevor Project, 2020, Wheldon et al., 2018), use disorders (Goldberg et al., 2013), and polysubstance use (Lee et al., 2023), compared to heterosexual YAs.

Prior research has examined common patterns of polysubstance use in YAs, such as low-level use (i.e., low probabilities of tobacco, cannabis, and alcohol use), tobacco and alcohol co-use, and polysubstance use (i.e., co-use of alcohol, cannabis, and illicit substances) (de Jonge et al., 2022). However, differences in patterns of tobacco, cannabis, and alcohol use by SM identity and gender remain largely unexplored. Prior work has largely aggregated across groups of YAs representing distinct sexual identities (e.g., bisexual and gay/lesbian) when examining polysubstance use disparities (Goodwin et al., 2022); however, disparities may vary. For example, bisexual women may be at greater risk relative to other populations (e.g., heterosexual women, lesbian women, SM men) due to a combination of gender-based discrimination (McCabe et al., 2010) and rejection from both heterosexual and SM communities (Callis, 2013). Research suggests greater disparities among SMYA women than among SMYA men (Goodwin et al., 2022), greater prevalence of tobacco, cannabis, and alcohol use among bisexual (versus lesbian or heterosexual) women (Romm et al., 2022, Schuler and Collins, 2020), and greater e-cigarette—but not combustible tobacco—use prevalence among bisexual versus lesbian women (Romm, Huebner, et al., 2022).

Tobacco, cannabis, and alcohol are the most commonly used substances in the US (Substance Abuse and Mental Health Services Administration, 2023) and may increase risk for leading causes of preventable death such as heart disease, cancer, respiratory disease, stroke, and unintentional injury (Yoon et al., 2014). Sexual identity disparities in tobacco, cannabis, and alcohol use first emerge in adolescence and young adulthood (Rosario et al., 2014, Schuler et al., 2018), presenting opportunity to intervene before long-term health consequences are incurred. Further research is needed to identify differential patterns of co-occurring combustible tobacco, e-cigarette, cannabis, and alcohol use among specific groups of SMYA women and men, to inform tailored interventions and messaging campaigns aimed at preventing problematic patterns of polysubstance use during this vulnerable period. The current study examined: 1) classes of polysubstance use (i.e., combustible tobacco, e-cigarette, cannabis, alcohol) via latent class analysis (LCA) and 2) associations among sexual identity (distinguishing bisexual, gay/lesbian, and heterosexual) and resulting classes among YA women and men, separately.

## 2 Methods

### 2.1 Participants and Procedures

This study analyzed survey data from a 2-year longitudinal study of YAs (aged 18–34) designed to examine correlates of cigarette and e-cigarette use among individuals recruited from 6 metropolitan statistical areas (MSAs: Atlanta, Boston, Minneapolis, Oklahoma City, San Diego, Seattle) with varied tobacco legislative contexts (Public Health Law Center, 2020). This study, detailed elsewhere (Berg et al., 2021), was approved by the George Washington University Institutional Review Board.

Participants were recruited in Fall 2018 and surveyed biannually until Fall 2020 (total of 5 waves). To recruit participants, ads posted on Facebook and Reddit targeted individuals by using indicators reflecting those eligible (i.e., ages 18–34, residing in one of the 6 MSAs, English-speaking). After clicking on an ad, individuals were directed to a webpage with a study description, consent form, and eligibility screener, then eligible individuals completed the online baseline survey via Alchemer. Upon completion, participants were asked to confirm their participation in the study by clicking a “confirm” link in an email sent a week later. Purposive, quota-based sampling ensured the sample represented sufficient numbers of cigarette and e-cigarette users (roughly one-third each), roughly equal numbers of women and men, and 40 % racial or ethnic minority individuals (subgroup enrollment was capped by MSA).

Of the 10,433 individuals who clicked on ads, 9,847 consented, of whom 2,751 (27.9 %) were excluded due to: (a) ineligibility (n = 1,472) and/or (b) their subgroup target being met (n = 1,279). Among the remaining 7,096 individuals, 48.8 % (n = 3,460) provided complete data, and 86.9 % (n = 3,006) confirmed participation (Berg et al., 2021). The current analyses involve the use of baseline sociodemographic data and Fall 2020 data (n = 2,476, 82.4 %), which assessed past-month use of tobacco products, cannabis, and alcohol. Among participants with baseline data, a greater proportion of those who did not complete vs. completed the Fall 2020 survey identified as male (47.9 % vs. 42.6 %, p = 0.030) and reported past-month combustible tobacco use (50.8 % vs. 32.1 %, p < 0.001), e-cigarette use (55.7 % vs. 33.8 %, p < 0.001), and cannabis use (51.2 % vs. 36.7 %, p < 0.001). Those who did not complete vs. completed the Fall 2020 survey were also younger (M = 24.06 vs. M = 24.66, p = 0.008). Fall 2020 completion was not associated with participant race, ethnicity, sexual identity, or past-month alcohol use.

### 2.2 Measures

#### 2.2.1 Stratification variable: gender

Participants were asked “What is your gender: male; female; or other?” Participants who selected “other” (n = 69) were excluded from primary analyses due to small sample size but explored in descriptive analyses.

#### 2.2.2 Primary correlate of interest: sexual identity

Participants were asked, “Do you consider yourself: heterosexual or straight; gay or lesbian; or bisexual?” These responses were recoded into dummy variables. Participants could also respond “other” (n *=* 43) or “prefer not to answer” (n = 37); participants who selected these options were excluded from primary analyses due to small sample sizes but were explored in descriptive analyses.

#### 2.2.3 Primary outcome: substance use

Participants reported the number of days they used each of the following products, separately, in the past 30 days (0–30 days): *combustible tobacco products* (i.e., cigarettes, cigars, hookah), *e-cigarettes, cannabis,* and *alcohol.* Those who reported ≥ 1 day of use were classified as a current user of each product; those who reported 0 days of use were classified as non-users of each product.

#### 2.2.4 Covariates

Sociodemographic covariates included age (continuous variable), race (categorized as White, Black, Asian, or another race due to group sizes), ethnicity (Hispanic vs. non-Hispanic), and education (< Bachelor’s degree vs. ≥ Bachelor’s degree). We also controlled for baseline depressive symptoms, which were assessed with the Patient Health Questionnaire – 2 item (PHQ-2), assessing symptoms in the past two weeks (0 = not at all to 3 = nearly every day). Participants with scores of > 3 were classified as having ≥ moderate depressive symptoms (α = 0.88) (Kroenke et al., 2003).

### 2.3 Data analysis

Descriptive statistics characterized the sample and screened for outliers. Latent class analyses (LCAs) of polysubstance use were fit in Mplus 8.8 and included 4 binary indicators (i.e., past-month combustible tobacco product, e-cigarette, cannabis, alcohol use). The best-fitting latent class solution was determined based on: 1) model interpretability; 2) lower Akaike information criterion (AIC), Bayesian information criterion (BIC), and sample-size adjusted Bayesian information criterion (aBIC) values, which indicate a better balance between model fit and parsimony; 3) higher entropy values, which indicate higher classification utility; and 4) a significant Lo-Mendel-Rubin (LMR) Adjusted Test (LRT), which suggests that the model is a significant improvement from a model with one fewer class. Model identification was confirmed with multiple sets of random starting values (500 for initial stage and 100 for final stage), with replication of the best log likelihood value. Next, bivariate analyses (i.e., ANOVAs, Chi-square tests) assessed associations among sexual identity and polysubstance use for women and men, separately. Finally, multinomial logistic regressions examined associations among sexual identity and polysubstance use class for women and men, separately, including the following covariates: age, race, ethnicity, education, and depressive symptoms. Prior to conducting regressions, we tested for multicollinearity of covariates and primary predictors. All tolerance and Variance Inflation Factor (VIF) values were > 0.25 (range = 0.92–0.97) and < 4 (range = 1.03–1.09), respectively, indicating that multicollinearity was not present (Kim, 2019). Missing data were minimal (<1% for cannabis use only) and were handled via full information maximum likelihood in Mplus when conducting LCA.

## 3 Results

### 3.1 Participant characteristics

Participants were 2,343 YAs aged 24.69 on average (SD = 4.70). Participants most commonly reported a heterosexual identity (72.6 %), followed by a bisexual identity (18.0 %), and a gay or lesbian identity (9.4 %). Regarding race and ethnicity, 70.8 % identified as White, 5.5 % Black, 13.3 % Asian, 10.4 % another race, and 11.1 % Hispanic. Most participants had ≥ a Bachelor’s degree (75.9 %) and 22.4 % reported ≥ moderate depressive symptoms.

Among women (57.4 %; *n =* 1,345), 24.8 % (*n =* 333) identified as bisexual, and 6.1 % (*n* = 82) as lesbian. On average, women were 24.65 years old (SD = 4.68), 7.1 % Black, 11.9 % Asian, 10.3 % another race, 9.9 % Hispanic; 77.2 % had ≥ a Bachelor’s degree, and 20.1 % reported ≥ moderate depressive symptoms. Past 30-day substance use rates were 20.1 % for combustible tobacco, 22.5 % for e-cigarettes, 42.8 % for cannabis, and 72.9 % for alcohol (Table 1***, left panel***).

**Table 1:** Bivariate analyses examining differences between participants identifying as heterosexual vs. gay/lesbian vs. bisexual, N = 2,343.

	**Women**				**Men**			
**Variables**	**Total** **N = 1,345 (100.0 %)**	**Heterosexual N = 930 (69.1 %)**	**Lesbian** **N = 82** **(6.1 %)**	**Bisexual N = 333 (24.8 %)**	**Total** **N = 998 (100.0 %)**	**Heterosexual N = 771 (77.3 %)**	**Gay** **N = 137 (13.8 %)**	**Bisexual** **N = 89** **(8.9 %)**
**Sociodemographics**								
Age, M (SD)	24.65 (4.68)	**24.92 (4.68)^a^**	**24.11 (4.49)^a,b^**	**24.02 (4.66)^b^**	24.74 (4.73)	**24.74 (4.81)^a^**	**25.57 (4.45)^a^**	**23.44 (4.25)^b^**
Race, N (%)								
White	951 (70.7)	**636 (68.4)^a^**	**62 (75.6)^a,b^**	**253 (76.0)^b^**	709 (71.0)	**530 (68.7)^a^**	**109 (79.0)^b^**	**70 (78.7)^a,b^**
Black	95 (7.1)	**78 (8.4)^a^**	**4 (4.9)^a,b^**	**13 (3.9)^b^**	34 (3.4)	25 (3.2)	6 (4.3)	3 (3.4)
Asian	160 (11.9)	127 (13.7)	4 (4.9)	29 (8.7)	151 (15.1)	**135 (17.5)^a^**	**10 (7.2)^b^**	**6 (6.7)^b^**
Other race	139 (10.3)	89 (9.6)	12 (14.6)	38 (11.4)	104 (10.4)	81 (10.5)	13 (9.4)	10 (11.2)
Hispanic, N (%)	133 (9.9)	85 (9.1)	8 (9.8)	40 (12.0)	128 (12.8)	97 (12.6)	17 (12.3)	14 (15.7)
Education, N (%)								
<Bachelor’s degree	307 (22.8)	**180 (19.4)^a^**	**22 (26.8)^a,b^**	**105 (31.5)^b^**	258 (25.9)	193 (25.0)	35 (25.4)	30 (33.7)
≥Bachelor’s degree	1038(77.2)	**750 (80.6)^a^**	**60 (73.2)^a,b^**	**228 (68.5)^b^**	740 (74.1)	578 (75.0)	103 (74.6)	59 (66.3)
**Depressive Symptoms, N (%)**								
<Moderate symptoms	1075 (79.9)	**777 (83.5)^a^**	**64 (78.0)^a,b^**	**234 (70.3)^b^**	744 (74.5)	**593 (76.9)^a^**	**98 (71.0)^a,b^**	**53 (59.6)^b^**
≥Moderate symptoms	270 (20.1)	**153 (16.5)^a^**	**18 (22.0)^a,b^**	**99 (29.7)^b^**	254 (25.5)	**178 (23.1)^a^**	**40 (29.0)^a,b^**	**36 (40.4)^b^**
**Past 30-day Substance Use, N (%)**								
Combustible tobacco	270 (20.1)	**169 (18.2)^a^**	**15 (18.3)^a,b^**	**86 (25.8)^b^**	299 (30.0)	**246 (31.9)^a^**	**26 (18.8)^b^**	**27 (30.3)^a,b^**
E-cigarettes	302 (22.5)	**173 (18.6)^a^**	**21 (25.6)^a,b^**	**108 (32.4)^b^**	286 (28.7)	**239 (31.0)^a^**	**24 (17.4)^b^**	**23 (25.8)^a,b^**
Cannabis	531 (42.8)	**304 (35.4)^a^**	**45 (58.4)^b^**	**182 (59.3)^b^**	399 (42.9)	**285 (38.8)^a^**	**62 (52.1)^b^**	**52 (66.7)^b^**
Alcohol	980 (72.9)	671 (72.2)	61 (74.4)	248 (74.5)	694 (69.5)	533 (69.1)	101 (73.2)	60 (67.4)
**Polysubstance Use Class, N (%)**								
Primarily-alcohol use	438 (32.6)	**351 (37.7)^a^**	**15 (18.3)^b^**	**72 (21.6)^b^**	253 (25.4)	**202 (26.2)^a^**	**39 (28.3)^a^**	**12 (13.5)^b^**
Polysubstance use	270 (20.1)	**169 (18.2)^a^**	**15 (18.3)^a,b^**	**86 (25.8)^b^**	299 (30.0)	**246 (31.9)^a^**	**26 (18.8)^b^**	**27 (30.3)^a,b^**
Non-use	247 (18.4)	180 (19.4)	15 (18.3)	86 (15.6)	178 (17.8)	131 (17.0)	29 (21.0)	18 (20.2)
Cannabis and alcohol co-use	238 (17.7)	**144 (15.5)^a^**	**26 (31.7)^b^**	**68 (20.4)^a,b^**	144 (14.4)	**93 (12.1)^a^**	**32 (23.2)^b^**	**19 (21.3)^b^**
E-cigarette, cannabis, and alcohol co-use	152 (11.3)	**86 (9.2)^a^**	**11 (13.4)^a,b^**	**55 (16.5)^b^**	124 (12.4)	99 (12.8)	12 (8.7)	13 (14.6)

*Note*. Bolded values denote statistically significant differences by sexual identity at p < 0.05. Values with different superscripts denote statistically significant post-hoc group differences at p < 0.05.

Among men (42.6 %; *n =* 998), 8.9 % (*n =* 89) identified as bisexual, and 13.8 % (*n* = 137) as gay. On average, men were 24.74 years old (SD = 4.73); 3.4 % were Black, 15.1 % Asian, 10.4 % another race, 12.8 % Hispanic; 74.1 % reported ≥ a Bachelor’s degree, and 25.5 % reported ≥ moderate depressive symptoms. Past 30-day substance use rates were 30.0 % for combustible tobacco, 28.7 % for e-cigarettes, 42.9 % for cannabis, and 69.5 % for alcohol (Table 1***, right panel).***

### 3.2 Polysubstance use classes

LCA indicated the 5-class model provided the best fit to the data (Table 2). Although AIC, BIC, and aBIC were lower for the 6-class than the 5-class model, the change in these values became minimal. Moreover, the 5-class model was selected over the 6-class model, as the 6-class model yielded 2 classes with identical probabilities across substance use indicators. Additionally, the 5-class model yielded an entropy value > 0.80 and 5 conceptually meaningful and distinct classes of sufficient sample size. Finally, the adjusted LMR-LRT test for the 5-class model was significant, indicating significant improvement in model fit for a model with 5 versus 4 classes.

**Table 2:** Model fit information and conditional probabilities of past 30-day substance use by latent class.

**Number of polysubstance use classes**	**AIC**	**BIC**	**aBIC**	**Entropy**	**Adjusted LMR-LRT** **p-value**
1	48407.07	48441.62	48422.56	–	–
2	46100.34	46169.45	46131.32	1.00	0<.001
3	43822.60	43926.27	43869.08	0.946	0<.001
4	41998.16	42136.38	42060.12	0.993	0<.001
5	41061.29	41234.07	41138.75	0.995	0<.001
6	40796.41	41003.74	40889.36	0.959	0<.001
7	40808.41	41050.30	40916.85	0.910	0.499
**Past 30-day substance use indicator**	Primarily-Alcohol Use, N = 691 (29.5%)	**Polysubstance Use,** **N = 569 (24.3 %)**	**Non-use,** **N = 425 (18.1 %)**	**Cannabis and Alcohol** **Co-Use, N = 382 (16.3 %)**	**E-cigarette, Cannabis, and Alcohol Co-Use,** **N = 276 (11.8 %)**
Combustible tobacco	0.00	1.00	0.00	0.00	0.00
E-cigarettes	0.00	0.55	0.00	0.00	0.98
Cannabis	0.00	0.56	0.18	0.99	0.57
Alcohol	1.00	0.71	0.00	1.00	0.72

*Note. The 6-class solution consisted of 2 classes with the same conditional probabilities (i.e., 2 classes reflecting primarily-alcohol use) and thus, the 5-class solution was chosen based on low AIC, BIC, and aBIC, high entropy, a significant adjusted LMR-LRT value, and theoretical interpretability.

As shown in Table 2***,*** the 5 classes include: 1) *Primarily-alcohol use* (*n =* 691, 29.5 %), consisting of individuals who displayed high probabilities for alcohol use, but low probabilities of combustible tobacco, e-cigarette, and cannabis use; 2) *Polysubstance Use* (*n* = 569, 24.3 %), with individuals who exhibited moderate to high probabilities of combustible tobacco, e-cigarette, cannabis, and alcohol use; 3) *Non-use* (*n* = 425, 18.1 %), characterized by individuals who displayed low probabilities of using all substances; 4) *Cannabis and alcohol co-use* (*n* = 382, 16.3 %), consisting of individuals who had high probabilities of cannabis and alcohol use, but low probabilities of combustible tobacco and e-cigarette use; and 5) *E-cigarette, cannabis, and alcohol co-use* (*n* = 276, 11.8 %), with individuals who displayed moderate to high probabilities of e-cigarette, cannabis, and alcohol use, but low probabilities of combustible cigarette use.

### 3.3 Associations between sexual identity and polysubstance use class among women

Most women were classified into the *Primarily-alcohol use* class (32.6 %), followed by *Polysubstance use* (20.1 %), *Non-use* (18.4 %), *Cannabis and alcohol co-use* (17.7 %), and *E-cigarette, cannabis, and alcohol co-use* (11.3 %). Bivariate analyses indicated that a smaller proportion of bisexual and lesbian (vs. heterosexual) women belonged to the *Primarily-alcohol use* class, whereas a larger proportion of bisexual (vs. heterosexual) women belonged to the *Polysubstance use* and *E-cigarette, cannabis, and alcohol co-use* classes. Additionally, a larger proportion of lesbian (vs. heterosexual) women belonged to the *Cannabis and alcohol co-use* class (Table 1***, left panel***). Multinomial logistic regression results indicated that, relative to heterosexual women, bisexual and lesbian women displayed lower odds for *Primarily-alcohol use*. Additionally, bisexual women displayed higher odds for *Polysubstance use* and *E-cigarette, cannabis, and alcohol co-use* (Table 3***, upper panel***).

**Table 3:** Multinomial logistic regression analyses predicting polysubstance use class among women and men (ref = Non-use).

	**Primarily-Alcohol Use**		**Polysubstance Use**		**Cannabis and Alcohol Co-Use**		**E-cigarette, Cannabis, and Alcohol Co-Use**	
**Variable**	**aOR**	**95 % CI**	**aOR**	**95 % CI**	**aOR**	**95 % CI**	**aOR**	**95 % CI**
**Women**								
Sexual identity (ref: Straight)								
Lesbian	**0.47**	**0.22, 0.98**	0.95	0.44, 2.03	1.93	0.97, 3.83	1.23	0.54, 2.85
Bisexual	**0.71**	**0.47, 0.97**	**1.52**	**1.10, 2.31**	1.52	0.98, 2.36	**1.78**	**1.11, 2.85**
Age	0.97	0.94, 1.01	1.03	0.99, 1.07	0.98	0.94, 1.03	0.97	0.92, 1.01
Race (ref: White)								
Black	**0.53**	**0.29, 0.95**	0.83	0.45, 1.55	**0.42**	**0.20, 0.88**	**0.31**	**0.11, 0.84**
Asian	**0.29**	**0.18, 0.45**	**0.39**	**0.23, 0.67**	**0.20**	**0.11, 0.36**	**0.31**	**0.16, 0.59**
Other	**0.59**	**0.35, 0.99**	0.59	0.33, 1.06	**0.53**	**0.29, 0.97**	0.69	0.36, 1.32
Hispanic	0.78	0.46, 1.32	0.85	0.48, 1.50	0.74	0.41, 1.36	0.49	0.23, 1.03
Education (ref: <Bachelor’s degree)								
≥Bachelor’s degree	**2.24**	**1.47, 3.39**	**0.64**	**0.43, 0.95**	**2.05**	**1.28, 3.29**	0.83	0.52, 1.32
Depressive symptoms (ref: <Moderate symptoms)								
≥Moderate symptoms	0.73	0.46, 1.14	**2.04**	**1.31, 3.18**	1.32	0.82, 2.14	**2.10**	**1.28, 3.46**
**Men**								
Sexual identity (ref: Straight)								
Gay	0.80	0.46, 1.39	**0.36**	**0.20, 0.66**	1.35	0.74, 2.44	**0.48**	**0.23, 0.99**
Bisexual	**0.43**	**0.20, 0.96**	0.69	0.35, 1.33	1.40	0.67, 2.90	0.82	0.38, 1.80
Age	1.00	0.95, 1.04	**1.07**	**1.02, 1.12**	1.01	0.96, 1.07	1.00	0.95, 1.05
Race (ref: White)								
Black	**0.22**	**0.08, 0.59**	**0.36**	**0.15, 0.88**	**4.53**	**2.32, 5.83**	0.43	0.14, 1.26
Asian	**0.32**	**0.19, 0.53**	**0.28**	**0.17, 0.48**	**0.22**	**0.11, 0.43**	**0.32**	**0.17, 0.61**
Other	0.77	0.37, 1.60	1.05	0.54, 2.04	0.67	0.29, 1.55	1.07	0.48, 2.39
Hispanic	0.87	0.45, 1.72	**1.91**	**1.05, 3.48**	0.70	0.32, 1.54	0.72	0.32, 1.63
Education (ref: <Bachelor’s degree)								
≥Bachelor’s degree	**2.26**	**1.36, 3.75**	0.68	0.44, 1.06	1.44	0.83, 2.48	0.93	0.55, 1.59
Depressive symptoms (ref: <Moderate symptoms)								
≥Moderate symptoms	0.53	0.33, 0.87	1.16	0.75, 1.78	0.66	0.38, 1.12	0.94	0.55, 1.59

*Note*. Bolded values denote statistically significant differences at p < 0.05.

### 3.4 Associations between sexual identity and polysubstance use class among men

Most men were classified into the *Polysubstance use* class (30.0 %), followed by *Primarily-alcohol use* (25.4 %), *Non-use* (17.8 %), *Cannabis and alcohol co-use* (14.4 %), and *E-cigarette, cannabis, and alcohol co-use* (12.4 %). Bivariate analyses indicated that a smaller proportion of bisexual (vs. heterosexual and gay) men belonged to the *Primarily-alcohol use* class. Additionally, a smaller proportion of gay (vs. heterosexual) men belonged to the *Polysubstance use* class and a larger proportion of gay and bisexual (vs. heterosexual) men belonged to the *Cannabis and alcohol co-use* class (Table 1***, right panel***). Multinomial logistic regression results indicated that, relative to heterosexual men, bisexual men displayed lower odds for *Primarily-alcohol use* and gay men displayed lower odds for *Polysubstance use* and *E-cigarette, cannabis, and alcohol co-use* (Table 3***, lower panel***).

### 3.5 Sub-analyses: “other” and “prefer not to answer” reports for sexual identity and/or gender

Of the 133 participants excluded from primary analyses, 43 (32.3 %) reported “other” for sexual identity (26 queer, 13 asexual, 4 not sure), and 37 (27.8 %) reported “prefer not to answer.” An additional 53 participants reported “other” for gender. Of those reporting “other” sexual identity, 25.6 % reported past-month combustible tobacco use, 30.8 % e-cigarette use, 43.6 % cannabis use, and 65.4 % alcohol use. Of those reporting “prefer not to answer” for sexual identity, 27.0 % reported past-month combustible tobacco use, 18.9 % e-cigarette use, 37.8 % cannabis use, and 62.2 % alcohol use. Of those reporting “other” gender, 23.2 % reported past-month combustible tobacco use, 26.1 % e-cigarette use, 53.6 % cannabis use, and 72.5 % alcohol use.

## 4 Discussion

This study identified 5 substance use patterns among YAs: primarily-alcohol use, polysubstance use (i.e., combustible tobacco, e-cigarette, cannabis, and alcohol use), non-use, cannabis and alcohol co-use, and e-cigarette, cannabis, and alcohol co-use. While primarily-alcohol use and polysubstance use were the most commonly endorsed patterns among both women and men, the profiles of substance use by gender and sexual identify subgroups differed significantly, underscoring the need for additional research identifying mechanisms and intervention targets to decrease high-risk substance use patterns for particular groups.

Consistent with prior research examining tobacco, cannabis, and alcohol use separately (Kerr et al., 2015, Romm et al., 2022, Schuler and Collins, 2020), this study found that bisexual (versus heterosexual) women were more likely to engage in polysubstance use, as well as e-cigarette, cannabis, and alcohol co-use. Bisexual women were less likely than heterosexual women to exhibit the most prevalent pattern—primarily-alcohol use. Greater risk for engaging in polysubstance use may occur for bisexual, but not lesbian women, as bisexual individuals often experience marginalization from both heterosexual and SM communities (Callis, 2013). Bisexual women, in particular, report feeling pressured to “prove” that they belong to the SM community (Cipriano et al., 2022). Among bisexual women—but not bisexual men—experiencing bisexual stigma from the SM community is associated with internalized stigma (Arriaga & Parent, 2019). Women who internalize bisexual stigma may use substances to cope (Hatzenbuehler, 2009, Meyer, 2003). For bisexual YAs, substance use serves functions such as facilitating connection with the SM community (e.g., smoking at parties) and relieving the stress of managing one’s identity differently in heterosexual versus SM social spaces (McQuoid et al., 2019). Targeted substance advertising is prevalent in SM social spaces and may partially account for substance use disparities (Belt et al., 2014, Stevens et al., 2004). SM women, particularly bisexual women (Tan et al., 2021), report greater exposure to tobacco and cannabis advertising than heterosexual women (Romm et al., 2024, Tan et al., 2021). Social spaces such as parties, where multiple substances are often available, may be conducive to polysubstance use for bisexual women who are struggling with bisexual stigma.

Compared to heterosexual men, gay men were less likely to engage in polysubstance use or in e-cigarette, cannabis, and alcohol co-use, and bisexual men were less likely to primarily use alcohol. Despite also being vulnerable to minority stress, bisexual men were less likely to use alcohol and gay men were less likely to engage in polysubstance use patterns characterized by combustible tobacco, e-cigarette, cannabis, and alcohol co-use, as well as e-cigarette, cannabis, and alcohol co-use, relative to heterosexual men. SM men’s lower likelihood of engaging in polysubstance use may be partially driven by their lower likelihood of tobacco use (Romm, Huebner, et al., 2022). In bivariate analyses, gay men displayed higher rates of cannabis and alcohol co-use – but not tobacco use – compared to heterosexual men, perhaps because YAs perceive cannabis-alcohol co-use as more effective for coping with stress than alcohol-only use (Boyle et al., 2021).

Minority stress experiences may be especially prevalent among individuals with multiple, intersecting minoritized identities (e.g., sexual identity, race, ethnicity) (Crenshaw, 1991). Black (versus White) men had greater odds of cannabis-alcohol co-use, and Hispanic (versus non-Hispanic) men had greater odds of polysubstance use. Importantly, however, several racially minoritized groups of participants (e.g., Asian and Black women and men) were *less* likely than their White peers to exhibit polysubstance use and co-use patterns. Findings underscore the complexity of intersecting identities and the need to disaggregate races, ethnicities, sexual identities, and genders when examining substance use patterns.

Taken together, findings have important implications for future research, policy, and practice. Findings urge researchers to examine specific sexual identity by gender subgroups, as well as the co-occurring use of a range of substances, and intersecting substance use disorders (e.g., dual diagnosis of cannabis and alcohol use disorders) among SMYAs. When weighing the public health impact of products such as e-cigarettes and cannabis, regulatory decision-makers should account for disparities in the prevalence of harmful polysubstance use patterns among SMYAs. Public health messaging campaigns and substance use intervention efforts should focus on the use of both combustible tobacco products and e-cigarettes along with cannabis and alcohol, tailoring efforts toward specific subgroups of SMYAs. Bisexual women, in particular, may benefit from psychoeducation regarding the risks of polysubstance use and from support in managing difficult experiences and emotions. Emotion regulation skills may be a fruitful target for engaging in harmful patterns of polysubstance use to cope with discrimination (Vogel, Romm, & Berg, 2024, Vogel, Romm, & Berg, 2024). Importantly, bolstering individuals’ resilience is not a substitute for reducing discrimination in society; however, building resilience may help SMYAs maintain their health and well-being.

### 4.1 Limitations and future directions

Results should be interpreted considering several limitations. First, use rates should not be interpreted as prevalence rates and findings should not be generalized to US YAs broadly, due to targeted recruitment from 6 MSAs. Second, response options for the survey item on gender identity collected in 2018 were “male,” “female,” and “other.” This terminology better reflects sex than gender, and we were unable to discern whether participants who selected “male” or “female” identified as cisgender or transgender. Relatedly, the subsamples of participants who selected “other” or “prefer not to answer” to describe their sexual and/or gender identity were too small to enable subgroup primary analyses. High substance use prevalence was observed in these groups, and future research should make a concerted effort to recruit more gender-expansive individuals and sexual minority individuals with non-LGB (i.e., not lesbian, gay, or bisexual) identities. Third, co-use was broadly defined as use of multiple substances in the past month (i.e., concurrent use) (Subbaraman & Kerr, 2015). Patterns in substance use may differ with alternative definitions of co-use (e.g., simultaneous use) (Klesges et al., 2011), and future research should explore patterns of both concurrent and simultaneous co-use by sexual and gender identity, as well as a wider range of substances (e.g., opioids). Fourth, we did not have data on potential minority stress mechanisms contributing to associations between sexual identity and polysubstance use patterns. Finally, we were unable to examine associations among specific sexual identity and changes in polysubstance use class membership over time due to limited power. YAs’ substance use in Fall 2020 may have been influenced by COVID-19. However, our prior work using this sample shows that sexual orientation was not associated with changes in cigarette, e-cigarette, alcohol, or cannabis use from before to during COVID-19 (Romm, Patterson, et al., 2022).

### 4.2 Conclusions

This study of YAs identified considerable diversity in substance use disparities between heterosexual and SM women and men. Bisexual women displayed the greatest disparities in multiple patterns of polysubstance use, which is concerning given established associations among polysubstance use and a range of longer-term health problems relative to single substance use. Addressing underlying factors driving polysubstance use among SM individuals, particularly bisexual women, is critical to inform tailored intervention and messaging campaign efforts.

## 5 Role of funding sources

This work was supported by the US National Cancer Institute (R01CA215155; PI: Berg). Dr. Vogel is supported by the National Institute on Drug Abuse (K01DA055073; PI: Vogel) and the National Institute of Mental Health (R21MH138954; PI: Vogel). Dr. Romm is supported by the National Institute on Drug Abuse (R01DA059480; PI: Romm), the National Institute on Minority Health and Health Disparities (R21MD019345; MPIs: Cohn, Romm), and the American Cancer Society (134128-IRG-19-142; PI: Romm). Drs. Vogel and Romm are also supported by the Oklahoma Tobacco Settlement Endowment Trust (TSET) contract #R22-03, and the National Cancer Institute grant awarded to the Stephenson Cancer Center (P30CA225520). Dr. Berg is also supported by other US National Institutes of Health funding, including the National Cancer Institute (R01CA278229, MPIs: Berg, Kegler; R21CA261884, MPIs: Berg, Arem; R01CA275066, MPIs: Yang, Berg), the National Institute on Drug Abuse (R01DA054751, MPIs: Berg, Cavazos-Rehg), the Fogarty International Center (R01TW012456, MPIs: Berg, Paichadze, Petrosyan), and the National Institute of Environmental Health Sciences/Fogarty (D43ES030927, MPIs: Berg, Caudle, Sturua). Funders had no role in the study design, collection, analysis or interpretation of the data, writing the manuscript, or the decision to submit the paper for publication.

## CRediT authorship contribution statement

**Erin A. Vogel:** Writing – original draft. **Katelyn F. Romm:** Writing – review & editing, Formal analysis, Conceptualization. **Carla J. Berg:** Writing – review & editing, Supervision, Funding acquisition, Conceptualization.

## Declaration of competing interest

The authors declare that they have no known competing financial interests or personal relationships that could have appeared to influence the work reported in this paper.

## Data Availability

Data will be made available on request.
